# Delusional Insanity

**Published:** 1897-04-03

**Authors:** Maurice Craig


### DELUSIONAL INSANITY.

Abstract of a Lecture (Post-Graduate Course) delivered at Bethlem Royal Hospital by Maurice Craig, M.A., M.B.Cantab, M.R.C.P.Lond.

Gentlemen,—Up to the present, we have been discussing the so-called affective or emotional forms of insanity. To-day you will have the opportunity of seeing patients suffering from delusional insanity, where the ideational side is affected in contra-distinction to the emotional. There is no hard and fast rule, as you have already seen, by which you can say that a case is delusional in type, or ought to be classed under melancholia, mania, &c., for it is often merely a question of degree. Some writers have classed delusional cases under three heads. Those with monomania of (1) unseen agency, (2) grandeur, (3) suspicion. But it is seldom that we see a true case of monomania, and even then it is not always a true case of delusional insanity, but rather the result of an acute attack of mania or melancholia. For us to rightly understand and be able to grasp the various delusions observed in varying cases of delusional insanity, it is necessary for us to remember that there are two factors concerned—(1) consciousness of self, (2) the consciousness of the relationship of one's self to surroundings. These may be affected in varying degrees, or the one may be disordered while the other is apparently normal. (1) Disorders of the consciousness of self are not uncommonly emotional, and really come under affective disorders, but I will just briefly refer to them. They may be subdivided into (*a*) disorders of the feeling of self, (*b*) disorders of the thought of self. To take the former first, there are those persons who have elevated and exaggerated feeling of well-being. They feel full of vigour and spirits, and out of this they develop delusions of superhuman strength and such-like ideas. Or another patient feels depressed and self-conscious. It is a new sensation to him, and he misconstrues the true state of affairs, questions himself why he should be different to others, and finally comes to a wrong conclusion, and the first seeds of delusion are sown. For we must remember that delusions, like everything else, have their beginning, and it takes months or even years for a delusion to become crystallised and fully organised. And herein lies the secret of treatment, for while the patient is full of doubts and suspicions, then is the time to attend to mind and body, and not to wait, as so many do, until delusions have become organised and fixed and until the patient has learnt to base his whole life and work it from that standpoint.

To return, other cases have merely an altered feeling of their consciousness of self; they are neither exalted nor depressed, but they say that they are "unnatural" or "different to what they used to be."

The next class of cases are those with disorders of the "thought of self," and this may be "general" or "partial." There is the classical case of Dr. Tuke's, who lost himself, and was found looking for himself under the bed. In these cases a new self seems to replace the old self, and it is under this head that those cases come who believe themselves to be animals, &c.

Then there are those persons who seem to have a double consciousness, and who live a separate life while in the two different states. The old self alternates with the new, and not uncommonly the new self knows nothing of the old self, neither does the old know the new. We had an interesting case of this condition two years ago.

Finally, there are those individuals in whom the old and the new self co-exist, and the patient believes himself to be two persons.

Cases of partial disorder of the knowledge of self are more common. Some persons retain to a large extent the knowledge of their identity, but they believe that some part of them is altered or changed. One gentleman we had here thought that he was full of chalk; others feel that a limb is growing large out of all proportion to its fellow, others that their brain has been removed, and that they cannot think; but, indeed, instances can be enumerated almost indefinitely.

The next large group of cases is that where the relation of self to surroundings is the basis of delusions. The patients may either consider that they themselves have great power, wealth, or influence over their surroundings, or that they are a source of evil to the community. On the other hand, a person may believe that society is conferring honours and position on him. Others, again, may persuade themselves that the world is against them, and that they are the victims of a systematised persecution. This last-named form constitutes a very large number of the cases of delusional insanity. They are commonly unstable individuals predisposed to insanity by heredity. The period of incubation is long and too commonly overlooked. They are solitary and shun others, as they believe themselves to be the object of ridicule. Then comes the period of insane interpretation. He sees sinister motives in the movements and gestures of others, but still everything is vague and undeveloped, it is only "somebody intends to do me some harm." Sensory hallucinations may now appear commonly as the result of expectancy, and in confirmation of his unorganised delusion. He then fixes on a certain society or group of individuals who are his persecutors. The case that you have just seen believes that the Vigilance Society are hunting him down. Slowly and steadily the delusion crystallizes out, and finally he may fix on one man as the originator and promoter of all his troubles. A patient so suffering may either be homicidal or suicidal, this varying with his character; for while one man will retaliate, another more peacably inclined will commit suicide rather than suffer a life-long persecution. There are those, again, who, out of delusions of persecution, develop ideas of grandeur. They ask themselves "Why is everybody so interested in me? It must be that I am some great personage." These cases are by no means uncommon.

The diagnosis of persecutory insanity is often far from easy, as the patient is not uncommonly clever, and will conceal his delusions. It is more by watching his mode of living and his behaviour in company that you can first learn that he is suspicious. Some individuals with an insane inheritage develop this form of insanity out of a natural disposition of pride or suspicion. Some physical infirmity may be the basis, such as deafness, and in these cases the prognosis is bad. Others, from some defection of personal appearance, skin disease and the like, may believe that they are shunned by society. In giving a prognosis one must first consider the length of time that the disorder has lasted, and to what state of development the delusions have reached. If definite delusions have lasted over a year, and the patient is apparently basing the whole conduct of his life upon it, then the prognosis is grave. Where there is a physical cause for the delusional condition the outlook must entirely depend upon the curability of the disease. Even if a person does not recover, as time goes on, there is a tendency for the delusions to grow weaker, and probably there is some mental enfeeblement, though this may not be marked, and is in no way in the same proportion as with disorders like acute mania. The patient becomes less aggressive, and may finally be able to again live at home with his friends.

Treatment should be commenced as soon as the state of affairs is diagnosed. I cannot warn you too seriously against delay in the treatment of these cases. A young man or woman who is constantly seeking solitude, who drops from time to time hints that people stare at them in the streets, or who show suspicion, maybe in a hundred little ways, should be at once properly treated. If the physical health is bad, restore that first, regulate the patient's life, and if possible move him from close office work. Outdoor exercise is of the utmost benefit. Encourage him in any hobby that may take his fancy: do not force society upon him, but steadily and firmly encourage him in mixing freely with his fellow-creatures. Allow him to look upon you as his friend, and never treat him otherwise than you would do a sane individual. If the trouble go from bad to worse, an institution is the only chance for recovery. It is no use trying to treat these cases in a private house for a number of reasons, one being that rules have obviously to be made for the individual, and must apparently savour of a personal nature, while in institutions the patient is only one among many who have to conform to regulation. Later on, these patients may be hostile and an institution staff has a good moral effect. Whether, it is to be a private or public asylum is, of course, entirely dependent upon the means at the command of the patient in question. Good food and a regular mode of living may in time be the means of restoring his mental state, but recovery is often long delayed. Where some physical disease is the basis of the delusional condition, this disease must be treated—for by so doing, as a rule, the mental improvement goes on *pari passu* with the physical improvement.

Such, gentlemen, is a brief account of delusional insanity; but allow me, in conclusion, to warn you that you may frequently have great initial difficulty in advising the patient's friends. The lay mind, in the first place, fails to recognise insanity in unsystematised delusions, and in the second place looks upon an asylum rather as a protection to society than as a home for the treatment of a suffering patient.

